# Data Mining in the Mixed Methods: Application to the Study of the Psychological Profiles of Athletes

**DOI:** 10.3389/fpsyg.2019.02675

**Published:** 2019-12-05

**Authors:** José L. Pastrana, Rafael E. Reigal, Verónica Morales-Sánchez, Juan P. Morillo-Baro, Rocío Juárez-Ruiz de Mier, José Alves, Antonio Hernández-Mendo

**Affiliations:** ^1^Department of Languages and Computer Science, University of Málaga, Málaga, Spain; ^2^University of Málaga, Málaga, Spain; ^3^Department of Social Psychology, Social Work, Anthropology and East Asian Studies, University of Málaga, Málaga, Spain; ^4^Department of Evolutionary Psychology and Education, University of Málaga, Málaga, Spain; ^5^Polytechnic Institute of Guarda, Guarda, Portugal

**Keywords:** data mining, clustering, sport, sport psychology, mixed methods

## Abstract

Data mining is seen as a set of techniques and technologies allowing to extract, automatically or semi-automatically, a lot of useful information, models, and tendencies from a big set of data. Techniques like “clustering,” “classification,” “association,” and “regression”; statistics and Bayesian calculations; or intelligent artificial algorithms like neural networks will be used to extract patterns from data, and the main goal to achieve those patterns will be to explain and to predict their behavior. So, data are the source that becomes relevant information. Research data are gathered as numbers (quantitative data) as well as symbolic values (qualitative data). Useful knowledge is extracted (mined) from a huge amount of data. Such kind of knowledge will allow setting relationships among attributes or data sets, clustering similar data, classifying attribute relationships, and showing information that could be hidden or lost in a vast quantity of data when data mining is not used. Combination of quantitative and qualitative data is the essence of mixed methods: on one hand, a coherent integration of result data interpretation starting from separate analysis, and on the other hand, making data transformation from qualitative to quantitative and 1 vice versa. A study developed shows how data mining techniques can be a very interesting complement to mixed methods, because such techniques can work with qualitative and quantitative data together, obtaining numeric analysis from qualitative data based on Bayesian probability calculation or transforming quantitative into qualitative data using discretization techniques. As a study case, the Psychological Inventory of Sports Performance (IPED) has been mined and decision trees have been developed in order to check any relationships among the “Self-confidence” (AC), “Negative Coping Control” (CAN), “Attention Control” (CAT), “Visuoimaginative Control” (CVI), “Motivational Level” (NM), “Positive Coping Control” (CAP), and “Attitudinal Control” (CACT) factors against gender and age of athletes. These decision trees can also be used for future data predictions or assumptions.

## Introduction

### Data Mining Added to Mixed Methods and Cluster Algorithms

Data mining is a technique that tries to find behavior patterns in large data sets in order to explain them. There are many data mining techniques. One of them is clustering. Clustering technique allocates data into subsets that share some characteristics. Elements in each cluster are or have some features similar to the other elements in the cluster, but they also are or have some features different to elements. This is a very useful tool because it allows you to find or identify unknown groups that frequently are not identified by humans (Zaki and Meira, 2014; Witten et al., 2016).

Cluster analysis is often used to learn about data distribution, raising common features of the data and noticing interesting clusters that can be analyzed in detail. Clustering is also taken as a previous step for several algorithms, such as “classification” or “attributes selection,” which would work better and faster over selected smaller set of attributes. Clustering is usually called automatic classification because it distributes data into sets (Dutt et al., 2015; Thomas et al., 2018).

Main clustering features are as follows:

• *Scalability*: Although many clustering algorithms can work reasonably well on small data sets with less than hundred elements, they work even better with a large database containing millions or even billions of elements like Web search results.
• *Ability to deal with different types of attributes*: As we cannot make any supposition about data, clustering algorithms are designed to work on numeric (interval-based) data, binary, nominal (categorical), ordinal data, or all of them.
• *Ability to deal with noisy data*: Data from real world has outliers, missing, unknown, or erroneous data, so clustering algorithms have to be strong and resilient to filter and reject the “noise” (Patel and Thakral, 2016).

In the following, the main clustering methods and their characteristics (Ramesh and Nandhini, 2017) will be described: (1) partitioning methods (look for mutually exclusive clusters of spherical shape; they are distance-based and frequently use the mean to represent the cluster center; they are effective for small to medium size data sets); (2) hierarchical methods (based on the idea that clustering is a hierarchical decomposition and cannot correct erroneous merges or splits; they may add other techniques like microclustering or see object “linkages”); (3) density-based methods (they look for arbitrarily shaped clusters; they work on the supposition that clusters are dense object zones in space separated by low-density zones; they set each point must have a minimum number of neighbor points to take outliers out); and (4) grid-based method (they put data into a grid that allows a fast processing dependent only on the size of the grid).

### Building a Classifier

A classifier identifies an instance’s class (Fayyad, 1996; Otero and Sánchez, 2006) based on a training set of data. WEKA is a software tool that implements some classifier algorithms. The “J48” algorithm has been used for data analysis in the current studio in order to generate decision trees.

The J48 decision tree algorithm is a classification tool (Kaur et al., 2015). It creates one acyclic graph structure (a tree) where attributes are represented in the internal nodes and the arcs represent how values are split. Each leaf node will be a value from goal class. Decision trees are often built from a training set and then they will be used as a model of the problem in order to predict a future behavior.

A frequently used technique in data mining is the decision tree learning. As previously mentioned, it will create a model based on one input training set of data that can be used to predict values in the target variable. Each leaf node represents one value of the goal class and each top-down route in the tree will be the decisions taken in the other variables of our study.

The decision tree algorithm involves three steps:

(1) One attribute I selected as target or goal class.
(2) Choose the attribute splitting data in the smallest number of subsets.
(3) Each generated node has to go to step #2 (Iterative Dichotomiser).

The ID3 algorithm was developed by Quinlan in 1986. It uses the concept of Information gain as its splitting criterion for splitting nodes. Root node will be the best predictor. In the following, the highest information gain attribute is selected as the splitting one.

It develops tree classifiers following three steps:

(1) Goal attribute is selected and its entropy is calculated.
(2) The characteristic with the highest information gain is taken.
(3) Build a node for that characteristic and repeat steps 1 and 2 for the built one until the ending criterion is obtained.

The ID3 decision algorithm uses two main concepts for tree building: entropy and information gain. Entropy will be the measure that represents the degree of randomness inside the set of data. So, entropy is zero when the sample is totally homogeneous; on the contrary, entropy will be one when the sample is absolutely uncertain.

### Sport and Gender

Sport is a way of having fun; it also helps to make relationships with other people and helps to develop psychological and social skills. However, in order to achieve that goal, it must be done in specific circumstances. Sportsmen should not be under immense pressure from coaches or parents, and physical requirements must be adapted to the features of the participants (DiFiori et al., 2018; Markati et al., 2018). In addition, the sport can help in other aspects such as moral development, leadership formation, or the promotion of prosocial behaviors. It is so important that, in those contexts, it works in such a way that sport can break social barriers due to aspects such as gender, race, or socioeconomic level (Misener and Mason, 2006).

In recent decades, there has been an increment of women’s participation in sport. Not just as athletes, but women have reached relevant roles in several fields such as technical teams or sports directions. Although there are still problems of gender discrimination in some sports disciplines, the progress made by women is clear (Gregg and Gregg, 2017; Carson et al., 2018). In fact, although women’s participation increased throughout the 20th century, women acceptance in traditional masculine sports did not occur until the last decades of the 20th century (Messner, 1988; Birrell and Theberge, 1994).

In recent years, researchers have been interested in the behavior and characteristics of both genders in the sport, as can be observed in multiple investigations (Hall and Oglesby, 2016; Schwesig et al., 2016; Gregg and Gregg, 2017). The advancement of female in sports has increased the number of studies carried out on women, finding jobs related to physiology, technical skills, or the psychology of sport (Eys et al., 2015; Kozina et al., 2016; Gross et al., 2018). Therefore, it is interesting to investigate the participation and behavior of women in sport, as well as to check the differences and similarities between both genders and their own characteristics.

### The Sports Performance Psychological Inventory (IPED)

The IPED tool was developed in Spain by Hernández-Mendo (2006). This questionnaire is an adaptation of the Psychological Performance Inventory (PPI) (Loehr, 1986, 1990). The study of psychological profile factors is important in sports psychology, because it can determine sports performance (Reigal et al., 2018a). This tool describes a series of skills that enable an athlete to cope with different tasks and circumstances during training and competition (Raimundi et al., 2016). Because of that, it is very important in sports, so the psychological skills training should be a part of the common training of athletes (Weinberg and Gould, 1999; Massuça et al., 2014).

The IPED (Hernández-Mendo, 2006; Hernández-Mendo et al., 2014) is a widely used tool to assess psychological skills in athletes. IPED has been used in sports such as triathlon, swimming, soccer, taekwondo, and beach-handball for male and female evaluations (e.g., López-Cazorla et al., 2015; González-Reyes et al., 2017; Martínez-Moreno, 2017; Reigal et al., 2018a). This questionnaire assesses the following factors: “self-confidence,” “negative coping control,” “attention control,” “visual-imagery control,” “motivational level,” “positive coping control,” and “attitude control” (Hernández-Mendo, 2006).

The main goal of this paper is to analyze more than 500 answers given with the IPED and develop a decision tree in order to find factors that influence gender and age in basketball players.

## Materials and Methods

### Participants

We have collected 10,944 records from the Sports Performance Psychological Inventory IPED survey where we have information about sport, age, etc., 51.59% of the record set were male and 48.41% were female (age: *M* = 24.97; *SD* = 7.32). Participants were users of MenPas^1^, which is an online psychosocial assessment platform (González-Ruiz et al., 2010, 2018). We try to set a relation between gender and basketball practice (592 records; 59.63% male and 40.37% female; age: *M* = 21.70; *SD* = 8.15).

### Instruments

The IPED tool (Hernández-Mendo, 2006; Hernández-Mendo et al., 2014) is a Spanish adaptation of the PPI (Loehr, 1986, 1990). It is used to evaluate several psychological skills used by athletes during competition. The IPED is one of the most widely used tools to evaluate psychological profiles in sports in recent years (e.g., Gómez-López et al., 2013; Morillo-Baro et al., 2016; Martínez-Moreno, 2017). It consists of 42 items, divided into the following dimensions: “self-confidence” (e.g., “I see myself as more like a loser than a winner in competition”), “negative coping control” (e.g., “I get angry and frustrated during competition”), “attention control” (e.g., “I become distracted and lose my focus during competition”), “visual-imagery control” (e.g., “Before a competition, I picture myself performing perfectly”), “motivational level” (e.g., “I am highly motivated to play my best”), “positive coping control” (e.g., “I can keep strong positive emotion flowing during competition”), and “attitude control” (e.g., “I am a positive thinker during competition”). The items are rated on a five-point Likert-type scale ranging from 1 (“almost never”) to 5 (“almost always”). The internal consistency values per dimension, again assessed by Cronbach’s alpha, were 0.77 for self-confidence, 0.66 for negative coping control, 0.61 for attention control, 0.81 for visual-imagery control, 0.78 for motivational level, 0.78 for positive coping control, and 0.80 for attitude control.

### Procedure

Participants took the IPED between 2016 and 2018 using the online psychosocial evaluation platform *MenPas* (González-Ruiz et al., 2010, 2018). Before, to be able to log in, any participant has to register him/herself. In order to complete the IPED, MenPas informs about instrument characteristics and instructions. In addition, contact information of researchers and managers of the computerized tool is shown to solve any doubt. Subsequently, researchers can log into the platform to gather the information generated.

Initially, we gathered 10,944 records from the Sports Performance Psychological Inventory IPED, but only 592 records were analyzed in this paper (people who played basketball). We also have classified data in order to get any relationship between IPED factors (“self-confidence,” “negative coping control,” “attention control,” “visual-imagery control,” “motivational level,” “positive coping control,” and “attitude control”) and the gender and age of people practicing that sport. Those relationships are modeled in J48 decision trees, which are a model of the data and also a tool to predict future data behaviors.

Helsinki Declaration principles were followed throughout the study (World Medical Association, 2013). In addition, participants authorized us to use their information for research when they have been registered in the platform. So, we obtained written and informed consent from them. The current study was approved by the Ethics Committee of the University of Málaga (19-2015-H). When it comes to minors, we have chosen to use at least one of the following procedures: (1) the parents or legal guardians will register and therefore sign the informed consent, or (2) if the minors are the ones who will register, the informed consent of the parents is requested.

### Data Analysis

The WEKA tool has been used for data mining analysis. WEKA stands for “Waikato Environment for Knowledge Analysis.” It has been developed in Java programming language and it has a lot of options for “data preprocessing,” “data classification,” “clustering,” “association rules,” and “visualization.” The saved archives can be used in several file formats such as ARFF (“attribute relation file format”), CSV (“comma separated values”), C4.5, and binary. Those archives can be loaded from one URL or from one SQL database using JDBC. One more option could be that data sources, classifiers, etc., are invoked as beans and they can be connected graphically (Srivastava, 2014).

Classification algorithms develop a model starting from a set of data. As aforementioned, decision tree algorithms allow us to build a model of the problem based on a training data set, which is easily understandable and which also allows us to predict a future behavior. We have used the WEKA J48 algorithm for data processing, which is an ID3 algorithm extension adding features such as pruning, working with missing values, managing continuous value attributes, etc.

The J48 classifier algorithm has been used in order to build our model. J48 generates a decision tree that represents a model of the problem based on supplied samples and covering the biggest amount of given samples (TP or true positive). A decision tree represents the information using tree-like graph decisions where nodes represent labeled classes and each branch represents one split subset (Moertini, 2003).

#### Algorithm Steps

(i) When every instance belongs to the same class, then that class labeled leaf is returned.
(ii) For each attribute, potential information must be calculated in order to calculate the gain in information that resulted.
(iii) Using that criterion, the best attribute is taken for branching.

#### Calculating Gain

“Entropy” is a data disorder measure that will be calculated for any sample ($\vec{y}$) as

$$E\,n\,t\,r\,o\,p\,y\,\left(\vec{y}\right)=-\sum_{j=1}^{n}\frac{\left|y_{i}\right|}{\left|\vec{y}\right|}\,\log\left(\frac{\left|y_{i}\right|}{\left|\vec{y}\right|}\right)$$

$$E\,n\,t\,r\,o\,p\,y\,\left(j|\vec{y}\right)=\left(\frac{\left|y_{i}\right|}{\vec{y}}\right)\,\log\left(\frac{\left|y_{i}\right|}{\vec{y}}\right)$$

Gain is calculated as

$$\text{Gain}\left(\vec{y},j\right)=|Entropy\left(\vec{y}\right)-Entropy\left(j|\vec{y}\right)|$$

The algorithm will maximize the gain, by dividing overall entropy because of split argument $\vec{y}$ by *j*.

#### Pruning

Pruning is a very useful step for taking outliers out. This will allow rejecting not well-defined data or those very different from neighbor data, which will decrease the number of classification errors and help obtain a more normal tree.

### Features of the Algorithm

One of the advantages of the algorithm is that it can handle either discrete or continuous attributes, or both attributes together. Continuous attributes are handled by selecting a threshold value of the attribute and splitting those that are less than, more than, or equal to it. In addition, this algorithm prunes the tree, removing branches that are not useful for reaching a leaf (goal node).

## Results

**FIGURE 1:**
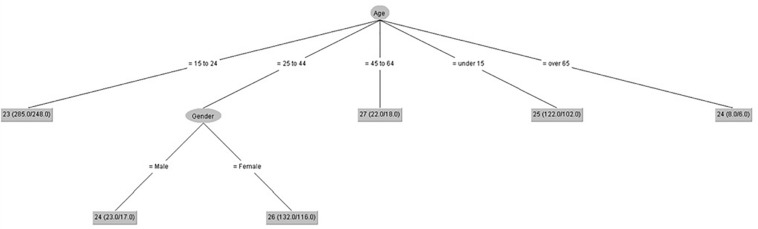
Self-confidence (IPED).

**FIGURE 2:**
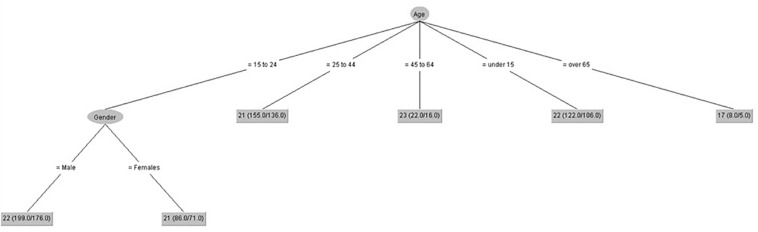
Negative coping control (IPED).

**FIGURE 3:**
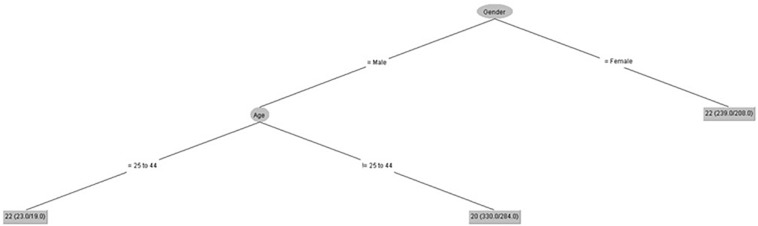
Attention control (IPED).

**FIGURE 4:**
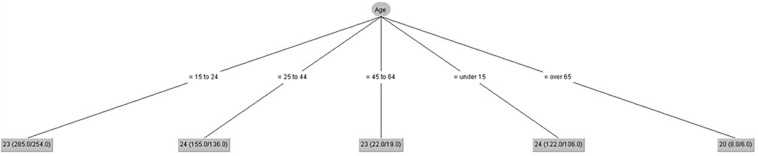
Visual-imagery control (IPED).

**FIGURE 5:**
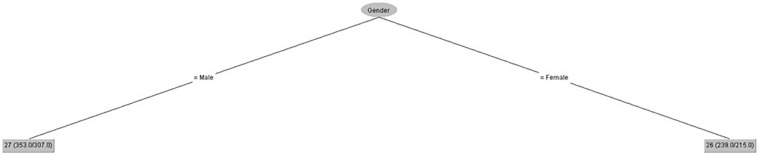
Motivational level (IPED).

**FIGURE 6:**
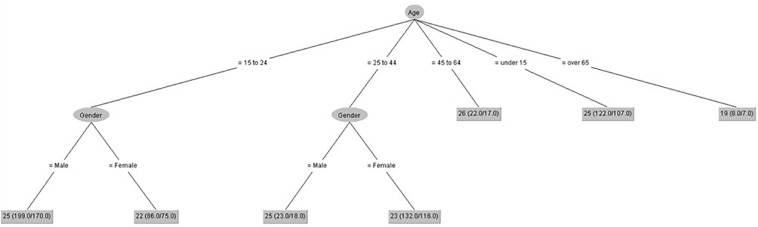
Positive coping control (IPED).

**FIGURE 7:**
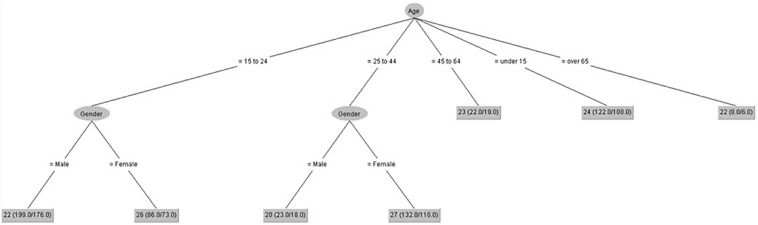
Attitude control (IPED).

We have developed seven decision trees (one for each IPED factor) (Figures 1–7), taking IPED factors as a goal to determine the influence of age and gender on IPED scores. Figure 1 shows that gender only had an influence on self-confidence in people from 25 to 44 years old, and the highest scores of self-confidence were in people from 45 to 64 years old.

Figure 2 shows that gender only had influence on negative coping control in people from 15 to 24 years old, and the lowest scores of negative coping control were in people over 65 years old.

Figure 3 shows that age only had an influence on attention control for male gender from 25 to 44 years old.

Figure 4 shows that gender had no influence on visual-imagery control. Age made some differences among groups. The highest scores were for people from 25 to 44 and people under 15 years old.

Figure 5 shows that gender had some influence on motivational level, but age had no influence.

Figures 6, 7 show that gender had some influence on people between 15 and 44 years old on positive coping control and attitude control. However, age had some influence on these scores. The lowest score on positive coping control was for people over 65 years. The lowest score on attitude control was for males between 25 and 44 years old.

## Discussion and Conclusion

This paper analyzed 592 answers given to the Sports Performance Psychological Inventory (IPED) and developed decision trees in order to find the influences of gender and age on IPED scores. Results showed that decision trees have allowed classifying the IPED factor scores according to gender and age. Data have shown differences by age and gender in all factors except motivational level (age) and visual-imagery control (gender). This shows that there are differences in sports psychological skills between men and women, and among young people, adults, and elderly. Because of this, in order to carry out more precise analyses of these constructs, the differences shown between the groups must be taken into account.

The techniques used have shown useful information on how the scores are distributed and they observe how the groups are classified. The results have shown great sensitivity to classify the data, observing different distributions by factor. The structure for each factor has been specific, which allows analyzing the behavior of each psychological skill evaluated with Sport Performance Psychological Inventory (IPED) by gender and age. This helps to make decisions about how to approach the interpretation of these dimensions for the studied sample. The findings suggest that these techniques are suitable to be applied in sports psychology to explore different variables that are usually studied in this field of knowledge.

Previous research has indicated that psychological skills of athletes can influence their performance, so it is interesting to analyze how they develop in athletes. Previous studies show how different psychological profiles have been observed (López-Cazorla et al., 2015; Martínez-Moreno, 2017; Reigal et al., 2018b). IPED scores change according to age, type of sport, or gender. Thus, it is interesting to obtain a score map structured by the variables that may be relevant in sports practice.

In the case study, we showed how data mining techniques could be applied to analyze and obtain information about quantitative and qualitative data in a set of data sample. Combination of quantitative and qualitative data analysis is the essence of mixed methods: on one hand, a coherent integration of result data interpretation starting from separate analysis, and on the other hand, making data transformation from qualitative to quantitative and vice versa (Cresswell and Plano Clark, 2011).

## Limitations and Future Works

This paper has some limitations that should be addressed in future work. On one hand, only basketball player answers have been analyzed. Future researches should evaluate other sports in order to check similarities and differences. In addition, other variables such as level or sporting experience could be included to observe the scores awarded. On the other hand, these analysis techniques have been sensitive with a heterogeneous sample. It would be interesting to determine if they effectively differentiate between more homogeneous groups according to specific sports variables (sports performance, competitive anxiety, minutes played, sports role, motivation, etc.).

## Data Availability Statement

The datasets generated for this study are available on request to the corresponding author.

## Author Contributions

AH-M, VM-S, JP, RR, JM-B, RJ-R, and JA participated in the study design and data collection, performed statistical analyses, contributed to the interpretation of the results, wrote the manuscript, approved the final manuscript as presented, and reviewed and provided feedback to the manuscript. JP, RR, and AH-M conceived the study and participated in its design and coordination. All authors made substantial contributions to the final manuscript.

## Conflict of Interest

The authors declare that the research was conducted in the absence of any commercial or financial relationships that could be construed as a potential conflict of interest.

## Footnotes

1 www.menpas.com
